# Overexpression of a ‘Beta’ MYB Factor Gene, *VhMYB15*, Increases Salinity and Drought Tolerance in *Arabidopsis thaliana*

**DOI:** 10.3390/ijms25031534

**Published:** 2024-01-26

**Authors:** Jiaxin Han, Jing Dai, Zhe Chen, Wenhui Li, Xingguo Li, Lihua Zhang, Anqi Yao, Bingxiu Zhang, Deguo Han

**Affiliations:** Key Laboratory of Biology and Genetic Improvement of Horticultural Crops (Northeast Region), Ministry of Agriculture and Rural Affairs, National-Local Joint Engineering Research Center for Development and Utilization of Small Fruits in Cold Regions, College of Horticulture & Landscape Architecture, Northeast Agricultural University, Harbin 150030, China; a02140301@163.com (J.H.); daijing@neau.edu.cn (J.D.); zhechen0313@163.com (Z.C.); lwh_neau@126.com (W.L.); xingguoli@neau.edu.cn (X.L.); zlh15009205209@163.com (L.Z.); a02200199@neau.edu.cn (A.Y.)

**Keywords:** grape, *VhMYB15*, salinity stress, drought stress

## Abstract

‘Beta’ is a hybrid of *Vitis riparia* L. and *V. labrusca* and has a strong ability to adapt to adverse growth environments and is mainly cultivated and used as a resistant rootstock. At present, the most extensively studied MYB TFs are R2R3-type, which have been found to be involved in plant growth, development, and stress response processes. In the present research, *VhMYB15*, a key transcription factor for abiotic stress tolerance, was screened by bioinformatics in ‘Beta’ rootstock, and its function under salinity and drought stresses was investigated. *VhMYB15* was highly expressed in roots and mature leave under salinity and drought stresses. Observing the phenotype and calculating the survival rate of plants, it was found that *VhMYB15*-overexpressing plants exhibited relatively less yellowing and wilting of leaves and a higher survival rate under salinity and drought stresses. Consistent with the above results, through the determination of stress-related physiological indicators and the expression analysis of stress-related genes (*AtSOS2*, *AtSOS3*, *AtSOS1*, *AtNHX1*, *AtSnRK2.6*, *AtNCED3*, *AtP5CS1*, and *AtCAT1*), it was found that transgenic *Arabidopsis* showed better stress tolerance and stronger adaptability under salinity and drought stresses. Based on the above data, it was preliminarily indicated that *VhMYB15* may be a key factor in salinity and drought regulation networks, enhancing the adaptability of ‘Beta’ to adverse environments.

## 1. Introduction

Plants in the natural environment are constantly challenged by changes in the environment, including abiotic and biotic stresses [1]. Salinity and drought are major threats to ecosystems, food security, and fruit quality [2]. Plants have evolved a series of regulatory mechanisms to cope with stress in the process of adapting to abiotic stress [3]. Studying the regulatory mechanisms of plant resistance to adversity is beneficial in selecting excellent resistant varieties [4]. In recent years, the function of transcription factors (TFs) in adversity has become a research hotspot [5].

The MYB family is divided into four subfamilies: 1R-MYB, 2R-MYB, 3R-MYB, and 4R-MYB according to the number of repeated R structures [6]. Different subfamily members perform different functions, among which, the 2R-MYB subfamily is the most studied. The N-terminal of the R2R3-MYB TF is composed of two R structures, while the C-terminal R structure has strong transcriptional activation function and certain plasticity, regulating protein activity. R2R3-MYB TFs play a core role in controlling plant metabolism, cell cycle and identification, development, and response to abiotic and biotic stresses [7]. Dicotyledonous and monocotyledonous plants have more than 100 R2R3-MYB members in their genomes. As of now, researchers have identified a large number of R2R3-MYB TFs from plants such as *Arabidopsis*, rice, and maize [8]. *AtMYB15*, an R2R3-MYB gene of *Arabidopsis*, responds to a variety of abiotic stresses [9]. *GsMYB15* is related to the survival ability of wild soybeans under salinity stress. *AtMYB49* improves the salt tolerance of *Arabidopsis* by activation of cutin deposition and antioxidant defense [10]. *GbMYB5* endows cotton and transgenic tobacco with drought tolerance [11], while *LcMYB1* endows transgenic *Arabidopsis* with salt tolerance [12]. 

R2R3-MYB TFs are identified using the RNA-Seq data of *Vitis vinifera* L. and 134 genes are obtained by collation and removal of redundancy [13]. Previously, the focus of attention on MYB TFs in grapes was mainly on the synthesis pathway of secondary metabolites [14]. *VvMYBPA1* in grapes is the first factor found to regulate their proanthocyanidins (PAs) synthesis [15], and its heterologous expression increases the synthesis of PAs in *Arabidopsis*. *VvMYBPA2* has high homology with *AtTT2*, and ectopic expression increases the biosynthesis of PAs in the hairy roots of grapes [16]. *VvMYB5a* and *VvMYB5b* genes act as positive regulators to impact the expression of structural genes controlling the biosynthesis of anthocyanin and proanthocyanidin [17,18]. In addition, MYB TFs are also associated with disease resistance in grapes. Transient expression of *VdMYB1* in *Vitis vinifera* promotes the expression of *VdSTS2* and enhances tolerance to grape powdery mildew [19]. However, little attention has been paid to the functions of R2R3-MYB TFs in grapes under salinity stress and drought stress, and there are also numerous gaps in the regulatory network. 

The ‘Beta’ grape is native to the United States and is a hybrid variety. It was introduced to China in the early years and has advantages such as drought tolerance, strong disease resistance, and cold tolerance. In the Heilongjiang region, salinity and drought problems are very serious. There are fewer varieties of grapes cultivated for processing. In addition to *Vitis amurensis Rupr*., ‘Beta’ can also be used for wine and juice. Therefore, ‘Beta’ can be used as a rootstock for grafting and raising seedlings. At the same time, it is also possible to cross ‘Beta’ with other grape varieties to obtain new varieties. So, it is necessary to study the pathways through which ‘Beta’ regulates salinity and drought tolerance. In this study, a salinity and drought stress-induced gene, *VhMYB15*, was screened from the genome of grapes. The aim of this study was to explore the valuable function of the MYB gene and provide a molecular basis for breeding rootstocks of resistant grapes.

## 2. Results 

### 2.1. Isolation and Bioinformatics Analysis of VhMYB15

Based on the homologous cloning of *VvMYB15* (XM_002285157.4, *Vitis vinifera* L.), the *VhMYB15* gene was obtained. The coding region of *VhMYB15*, from the initiation codon to the termination codon, contained a total of 762 nucleobases and was composed of 253 amino acids, of which Ser (11.1%), Leu (7.9%), and Glu (7.5%) accounted for a large proportion (Figure S1). Predictive analysis of the VhMYB15 protein revealed that its molecular weight and isoelectric point were 28.42 kDa and 5.42, respectively. Since the average hydrophilic coefficient was −0.675, VhMYB15 was a hydrophilic protein.

**Figure 1:**
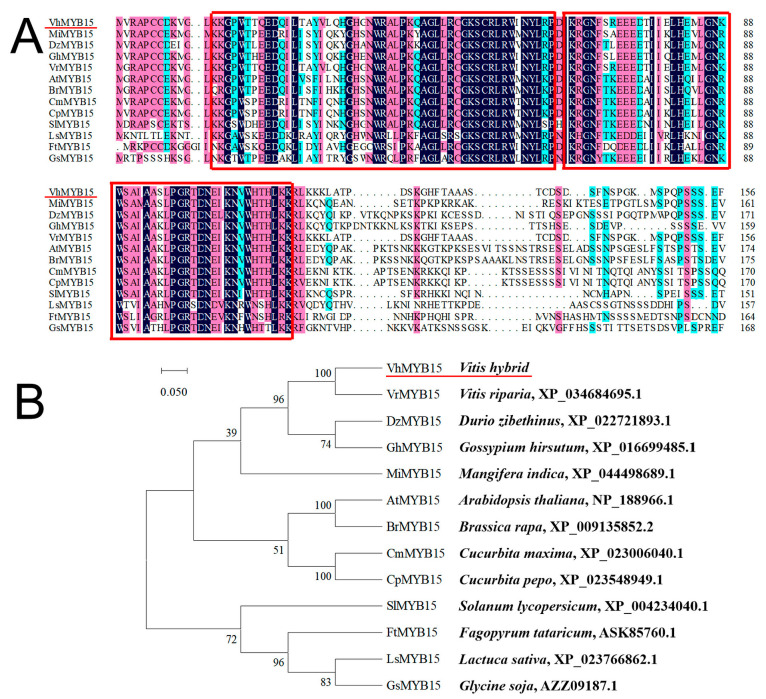
Multiple sequence analysis of VhMYB15 protein. (**A**) MYB sequence alignment. (**B**) Evolution tree. The red line and red box represent the target protein and R repeat sequence, respectively.

By predicting and analyzing the secondary structure of the VhMYB15 protein, it was found that it consisted of 30.83% alpha helices, 4.74% beta turns, 4.35% extended strands, and 60.08% random coils (Figure S2A). VhMYB15 contained two SANT conserved domains at 13–63 aa and 66–114 aa (Figure S2B). Predicting the tertiary structure of VhMYB15, it was found that the predicted model was consistent with the prediction of the secondary structure and the prediction of the conservative domain (Figure S2C). Multiple sequence analysis revealed that VhMYB15, like MYB proteins from other species, contained R2 and R3 domains (Figure 1A). Analyzing the evolutionary relationship, it was found that VhMYB15 and VrMYB15 (*Vitis riparia* L.) clustered on the same branch, with the closest evolutionary relationship. In addition, together with DzMYB15, GhMYB15, and MiMYB15, they formed the first cluster of the evolutionary tree. AtMYB15, BrMYB15, CmMYB15, and CpMYB15 constituted the second cluster, which was also closely related to the evolution of VhMYB15. The third cluster, consisting of SlMYB15, FtMYB15, LsMYB15, and GsMYB15, was relatively distant (Figure 1B). 

### 2.2. Subcellular Localization of VhMYB15 Protein 

**Figure 2:**
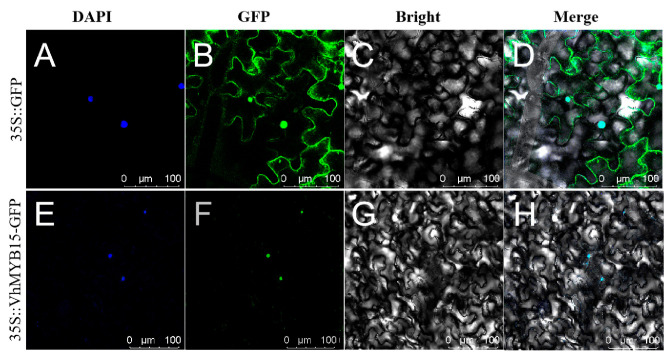
Subcellular localization of VhMYB15. The image consists of four fields of view: GFP, DAPI, Bright, and Merge. (**A**–**D**) 35S::GFP field of view. (**E**–**H**) 35S::VhMYB15-GFP field of view. Bar = 100 μm.

By injecting the fusion vector *VhMYB15*–pCAMBIA1300 containing a GFP tag into the leaves of *Nicotiana benthamiana*, protein localization was determined. Combining the fluorescence of DAPI and GFP, it was found that green fluorescence was distributed throughout the entire cell of tobacco injected with the 35S::GFP vector. In tobacco injected with the 35S::VhMYB15-GFP vector, the green fluorescence coincided with the blue fluorescence of DAPI, so it was determined that VhMYB15 was localized in the nucleus (Figure 2).

### 2.3. Analysis of the Expression Characteristics of VhMYB15 in ‘Beta’

**Figure 3:**
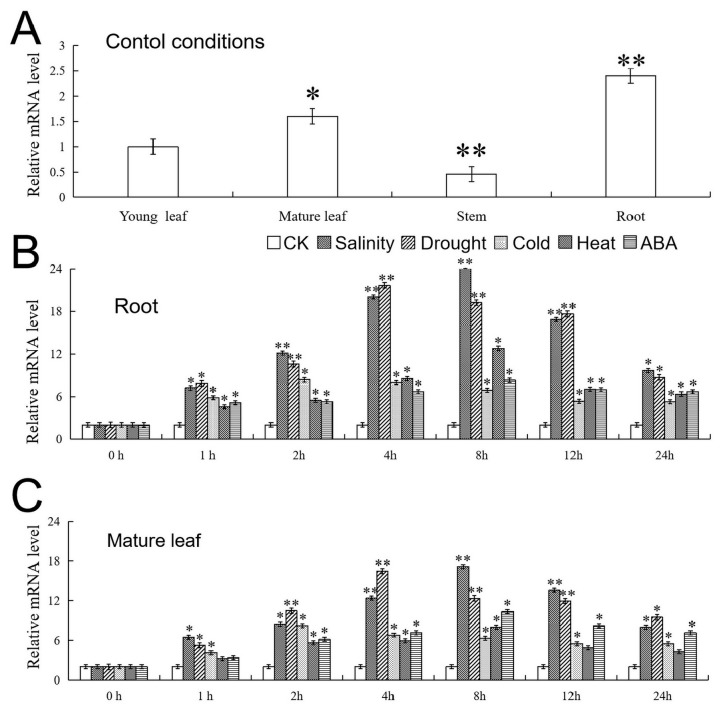
The expression of *VhMYB15* in ‘Beta’. (**A**) Expression of *VhMYB15* in different parts. The expression levels of *VhMYB15* in young leaves as a control. (**B**) The relative expression of *VhMYB15* in roots and (**C**) mature leaves under CK and stress treatments, with the expression level under CK conditions as the control. The SD is represented by an error bar (*n* = 3). (*) *p*-value ≤ 0.05, (**) *p*-value ≤ 0.01.

The tissue-specific expression characteristics of *VhMYB15* in ‘Beta’ were analyzed by qRT-PCR. The data showed that the expression level of *VhMYB15* was high in roots, relatively high in mature leaves, and low in stems and young leaves, indicating that the main function of *VhMYB15* may be nutrient transport (Figure 3A).

Salt, drought, cold, heat, and ABA stresses can all induce the expression of *VhMYB15*. Within 24 h of stresses, the changes in expression levels under different stresses showed a single-peak pattern. Data analysis found that under salt, drought, cold, heat, and ABA stresses, the time points at which the expression peaks occurred in roots and leaves were the same, 8 h, 4 h, 2 h, 8 h, and 8 h, respectively. In addition, these results also indicated that the up-regulation of *VhMYB15* expression was more induced by salt and drought stresses (Figure 3B,C).

### 2.4. Heterologous Expression of VhMYB15 in Arabidopsis Improved Salinity Tolerance

**Figure 4:**
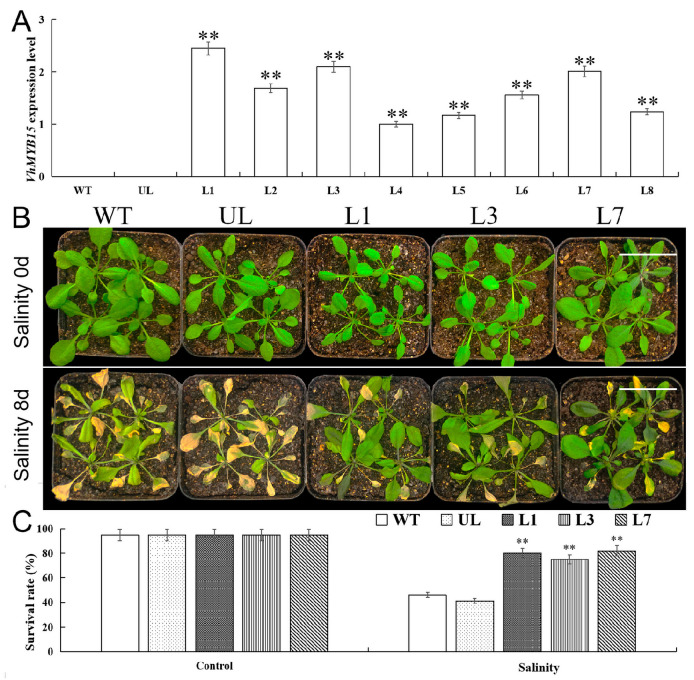
*VhMYB15*-overexpressing *Arabidopsis* increased salinity tolerance. (**A**) The expression of *VhMYB15* in *Arabidopsis* in different lines (wild type: WT, unloaded line: UL, transgenic lines: L1–8). (**B**) Phenotypes of WT, UL, L1, L3, and L7 *Arabidopsis* at different stages (Salinity 0 d, Salinity 8 d). Bar = 3 cm. (**C**) Survival rates of *Arabidopsis* at different salinity stages. Using WT as control. The SD is represented by an error bar (*n* = 3). (**) *p*-value ≤ 0.01.

To analyze whether *VhMYB15* plays a regulatory role in salinity and drought stresses, a *VhMYB15*–pCAMBIA1300 fusion vector was constructed. Transgenic plants were obtained by transforming the pCAMBIA1300 vector and *VhMYB15*–pCAMBIA1300 vector into *Arabidopsis*. RNA extraction was performed on plants with positive kanamycin screening for qRT-PCR analysis. *VhMYB15* was not expressed in the wild type (WT) or unloaded line (UL), while *VhMYB15* was expressed in positive plants to varying degrees, indicating the successful transfer of *VhMYB15* in *Arabidopsis* (Figure 4A). From the eight transgenic lines (L1, L2, L3, L4, L5, L6, L7, and L8), L1, L3, and L7, lines with high expression of *VhMYB15*, were selected to continue cultivation for the next experimental analysis.

In order to analyze the effect of *VhMYB15* on the tolerance of *Arabidopsis* to salinity stress, all lines of *Arabidopsis* were treated with 200 mM NaCl to analyze the effects of stress on plant phenotype and survival rate. Under control conditions (Salinity 0 d), the growth trend of *Arabidopsis* in all lines was basically the same, and there was no significant difference in survival rate. After 10 d of salinity treatment (Salinity 10 d), both *VhMYB15*-overexpressing (L1, L3, L7) and control-group plants (WT and UL) exhibited yellowing in their leaves, and there were obvious differences in phenotypes. *VhMYB15*-overexpressing *Arabidopsis* leaves had a lighter yellowing phenomenon (Figure 4B). After relieving the stress conditions and returning to normal growth for 3 d, it was found that the survival rates of *VhMYB15*-overexpressing *Arabidopsis* (80%, 75%, and 82%) were significantly higher than those of the control group *Arabidopsis* (46% and 41%) (Figure 4C).

**Figure 5:**
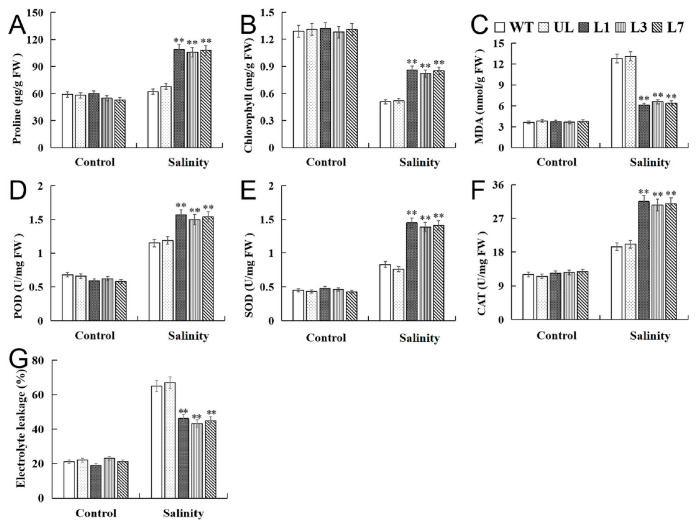
Analysis of proline (**A**), chlorophyll (**B**), MDA (**C**), POD (**D**), SOD (**E**), CAT (**F**), and electrolyte leakage (**G**) in *Arabidopsis* (wild type: WT, unloaded line: UL, transgenic lines: L1, L3, L7) at different salinity stages (Salinity 0 d, Salinity 8 d). Using WT indicators as controls. The SD is represented by an error bar (*n* = 3). (**) *p*-value ≤ 0.01.

Under control conditions (Salinity 0 d), there was no obvious difference in the physiological indicators of all *Arabidopsis* lines, but the physiological indexes changed under salinity stress, showing obvious differences. Salinity treatment resulted in an increase in plant antioxidant enzyme activity, proline content, MDA content, and electrolyte leakage. The activities of CAT, SOD, POD, and proline content of *VhMYB15*-overexpressing *Arabidopsis* were the highest (Figure 5A,D–F), while MDA content and electrolyte leakage were the lowest (Figure 5C,G). In addition, the content of chlorophyll was also affected by salinity stress and decreased, but the *VhMYB15*-overexpressing *Arabidopsis* was less affected (Figure 5B). The measured physiological indicators of plants before and after salinity stress further indicate that overexpression of *VhMYB15* may enhance plant tolerance to salinity stress.

### 2.5. VhMYB15 in Transgenic Arabidopsis Activated Salinity Tolerance-Related Genes

**Figure 6:**
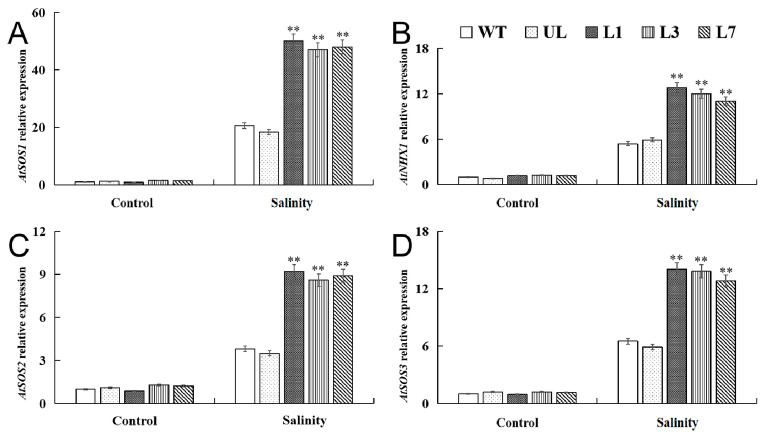
qRT-PCR detection of salinity tolerance-related gene expression in *Arabidopsis* (wild type: WT, unloaded line: UL, transgenic lines: L1, L3, L7). Relative expression levels of (**A**) *AtSOS1*, (**B**) *AtNHX1*, (**C**) *AtSOS2*, and (**D**) *AtSOS3* at different salinity stages (Salinity 0 d, Salinity 8 d). Using WT as control. The SD is represented by an error bar (*n* = 3). (**) *p*-value ≤ 0.01.

The expression levels of *AtSOS1*, *AtSOS2*, *AtSOS3*, and *AtNHX1* genes were analyzed by qRT-PCR. The results showed that the expression levels of *AtSOS1*, *AtSOS2*, *AtSOS3*, and *AtNHX1* did not differ significantly among all lines of *Arabidopsis* when not subjected to salinity stress. Salinity stress induced the expression of these responsive genes, and the expression level in *VhMYB15*-overexpressing *Arabidopsis* was higher. Therefore, we speculate that *VhMYB15* may alleviate osmotic pressure and maintain the balance of intracellular Na^+^ concentration by activating the expression of salt stress-responsive genes, thereby enhancing plant tolerance to salinity stress (Figure 6).

### 2.6. Heterologous Expression of VhMYB15 in Arabidopsis Improved Drought Tolerance

**Figure 7:**
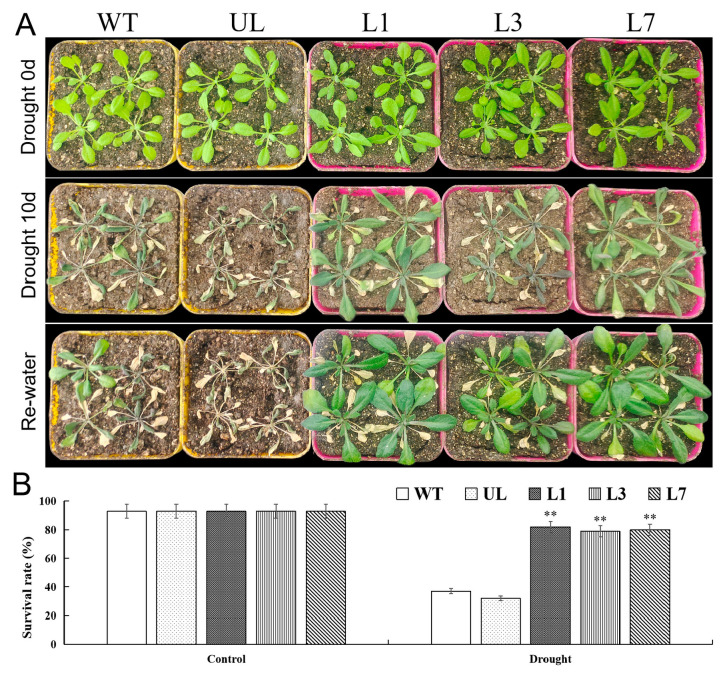
*VhMYB15*-overexpressing *Arabidopsis* increased drought tolerance. (**A**) Phenotypes of wild type (WT), unloaded line (UL), and transgenic lines (L1, L3, L7) *Arabidopsis* at different stages (Drought 0 d, Drought 10 d, Re-water). Bar = 3 cm. (**B**) Survival rates of *Arabidopsis* at different drought stages. Using WT as control. The SD is represented by an error bar (*n* = 3). (**) *p*-value ≤ 0.01.

Under control conditions (Drought 0 d), overexpression of *VhMYB15* did not cause phenotypic differences in *Arabidopsis*, and all lines had good leaf growth, with green color and no yellowing. After 10 d of water deficiency (Drought 10 d), phenotypic differences between *VhMYB15*-overexpressing *Arabidopsis* and the control-group *Arabidopsis* appeared (Figure 7A). Water scarcity leaded to leaf withering, while overexpression of *VhMYB15* weakened this trend. After watering again and returning to normal conditions for 3 d, most of the *VhMYB15*-overexpressing *Arabidopsis* resumed growth, with survival rates of 82%, 79%, and 80%, while most of the control-group *Arabidopsis* died (Figure 7B).

**Figure 8:**
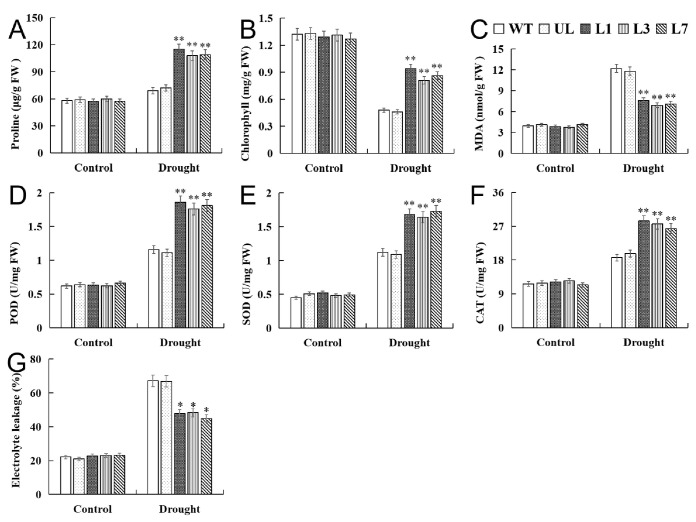
Analysis of proline (**A**), chlorophyll (**B**), MDA (**C**), POD (**D**), SOD (**E**), CAT (**F**), and electrolyte leakage (**G**) in *Arabidopsis* (wild type: WT, unloaded line: UL, transgenic lines: L1, L3, L7) at different drought stages. Using WT indicators as controls. The SD is represented by an error bar (*n* = 3). (*) *p*-value ≤ 0.05, (**) *p*-value ≤ 0.01.

Under control conditions (Drought 0 d), there was no significant difference in physiological indicators among all *Arabidopsis* lines, but physiological indicators changed under drought stress, and there were significant differences. The water shortage resulted in increases in proline content, MDA content, and electrolyte leakage and decrease in chlorophyll content (Figure 8A–C,G). In addition, the activity of antioxidant enzymes was also improved. The activities of CAT, SOD, and POD and the contents of proline and chlorophyll in *VhMYB15*-overexpressing *Arabidopsis* were significantly higher than those in the control group, while MDA content was the opposite, significantly lower than that of the control group (Figure 8D–F). 

### 2.7. VhMYB15 in Transgenic Arabidopsis Activated Drought Tolerance-Related Genes

**Figure 9:**
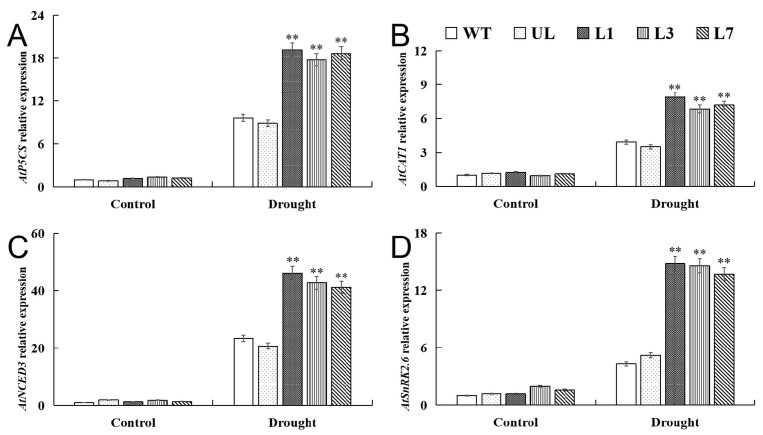
qRT-PCR detection of drought tolerance-related gene expression in *Arabidopsis* (wild type: WT, unloaded line: UL, transgenic lines: L1, L3, L7). Relative expression levels of (**A**) *AtP5CS*, (**B**) *AtCAT1*, (**C**) *AtNCED3*, and (**D**) *AtSnRK2.6* at different drought stages. Using WT as control. The SD is represented by an error bar (*n* = 3). (**) *p*-value ≤ 0.01.

We conducted qRT-PCR analysis of the expression levels of the key enzyme gene for proline synthesis *P5CS1*, the catalase 1 (*CAT1*) gene, the key enzyme in ABA synthesis gene *NCED3*, and the key gene *SnRK2.6* that regulates stomatal closure in *Arabidopsis*. The results showed that under sufficient irrigation conditions, there was no significant difference in the expression levels of *AtP5CS1*, *AtCAT1*, *AtCAT1*, and *AtSnRK2.6* among various lines of *Arabidopsis*. The expression of these genes increased due to dehydration, and the expression of *VhMYB15*-overexpressing *Arabidopsis* was significantly higher than that of the control group (Figure 9). 

## 3. Discussion

‘Beta’ is often used as a hybrid parent or grafting stock to obtain varieties with better tolerance and quality. At the same time, ‘Beta’ is also used as a processing variety for brewing and making juices [20]. MYB TFs are widely present in animals, plants, and fungi and are also one of the largest TF families in plants, possessing multiple biological functions [21]. It is not only involved in the regulation of plant growth and development [22,23], but it also participates in plant tolerance to stresses such as drought, salinity, and cold [24,25,26]. Therefore, it is necessary to study the function of MYB TFs in ‘Beta’, which is beneficial for grape breeding using genetic engineering technology. In this research, the *VhMYB15* gene was cloned and isolated from ‘Beta’ using *VvMYB15* (XM_002285157.4, *Vitis vinifera* L.) as a homologous gene. The VhMYB15 protein shared the same conserved domain with MYB15 proteins from other species and contained two SANT-MYB DNA binding domains, belonging to the typical R2R3-MYB protein (Figure 1).

TFs typically perform transcriptional regulatory functions in the nucleus, so most TFs are located at the subcellular level in the nucleus, and nuclear localization has gradually become the standard for identifying genes as TFs [27]. In this study, the 35S::VhMYB15-GFP vector was constructed and introduced into tobacco cells, and it was observed that it was also located in the nucleus, consistent with previous studies and consistent with the general characteristics of TFs.

MYB TFs are involved in regulating plant tolerance to a variety of adverse environments. For example, the expression of *GhMYB113* is positively regulated by drought stress and negatively regulated by salt stress [28]. *ZmMYB002* is highly expressed in maize seeds, roots, and leaves and can be significantly induced by drought and salt stresses [29]. *VvMYB112* and *VvMYB15* are induced by salt, drought, and cold, while *VvMYB107* and *VvMYB87* are only induced by salt and are not sensitive to drought or cold [30]. This study used qRT-PCR to detect the expression of *VhMYB15* in different tissue parts, with tissue specificity and the highest expression level observed in the roots (Figure 3A). Therefore, the roots can give priority to responding to adverse conditions. In addition, *VhMYB15* was expressed in both leaves and roots under salinity, drought, cold, heat, and ABA stresses, but its expression patterns were different. The response rate of *VhMYB15* to salinity and drought stresses was relatively rapid, and the expression of *VhMYB15* decreased rapidly with the extension of stress time, with a large change range. Under cold, heat, and ABA stresses, the expression trend of *VhMYB15* was the same as that of salinity and drought, but the expression level was relatively low (Figure 3). Therefore, salinity and drought were selected as stress treatment conditions for further analysis of *VhMYB15* function.

When plants are subjected to salt and drought stresses, the balance of REDOX reactions within their cells is disrupted. This imbalance can lead to the production of a large number of reactive oxygen species (ROS) and other harmful metabolites in plants [31]. These substances accumulate to a certain extent, which can damage the cellular structure of plants, increase the permeability of cell membranes, and lead to a large amount of electrolyte leakage [32]. In addition, ROS can also inhibit the activity of biological enzymes, thereby affecting various physiological and metabolic reactions of plants. 

Plants can eliminate reactive ROS free radicals through POD, SOD, and CAT, reduce cell membrane peroxidation, alleviate cell membrane osmotic pressure, and improve survival ability under adverse conditions [33,34]. Salt and drought stress accelerate the degradation of chloroplasts in plants, leading to a decrease in the stability of thylakoid membranes and a significant decrease in chloroplast absorption of light energy, ultimately leading to a decrease in photosynthetic rate [35]. MDA is the end product of membrane lipid peroxidation [36], and its changes in content can reflect the degree of membrane system damage. Stress can trigger or exacerbate membrane lipid peroxidation in plant cells and increase plasma membrane permeability and MDA content, leading to the worsening of biofilm damage [37]. In addition, plants also alleviate stress damage through solute accumulation, and some studies suggest that proline is the most effective osmotic regulator [38]. By observing the phenotype of *VhMYB15*-overexpressing *Arabidopsis* and measuring related physiological indicators, the function of *VhMYB15* under salinity and drought stresses was analyzed. It was found that *VhMYB15*-overexpressing *Arabidopsis* suffered significantly less damage under salinity and drought stresses than the control group, resulting in better growth and higher survival rate after stress relief (Figure 4 and Figure 7). Under salinity and drought stresses, the antioxidant enzyme activity, chlorophyll content, and proline content of *VhMYB15*-overexpressing *Arabidopsis* was significantly higher than that of the control group, while MDA content and electrolyte leakage were the opposite, which was consistent with the phenotype of *Arabidopsis* (Figure 5 and Figure 8).

We verified the difference in tolerance between *VhMYB15*-overexpressing *Arabidopsis* and control *Arabidopsis* under stress at the molecular level. The *AtSOS* pathway is currently one of the most extensively studied mechanisms by which plants regulate salt stress responses [39]. *SOS1*, *SOS2*, and *SOS3* constitute the SOS signaling pathway for salt tolerance in *Arabidopsis*, playing an important role in regulating K^+^/Na^+^ homeostasis in plants under salt stress [40]. The *AtSOS1* encodes a Na^+^/H^+^ reverse transporter protein, mediating Na^+^ efflux and transport and protecting cells from Na^+^ toxicity [41]. The *AtSOS2* gene encodes serine/threonine protein kinases, and its activity depends on the regulation of the calcium-binding protein AtSOS3 [42]. AtSOS3 activates and interacts with the AtSOS2 protein kinase [43]. The phosphorylation of the AtSOS3–AtSOS2 complex can activate the AtSOS1 protein, directly promoting Na^+^/H^+^ exchange controlled by AtSOS1 [44]. In addition, it may also negatively regulate the process of Na^+^ entering cells. *NHX1* (Na^+^/H^+^ antagonist, NHX) encodes the Na^+^/H^+^ reversal transporter located on the vacuole membrane [45]. It can transport excess Na^+^ within the cell to the vacuole, thereby reducing Na^+^ accumulation in the cytoplasm and improving salt tolerance [46]. Overexpression of *IlNHX* in tobacco maintains a high K^+^/Na^+^ ratio in tissues under salt stress, while reducing chlorophyll loss and lipid peroxidation, thereby improving tobacco salt tolerance [47]. Our research results were consistent with the expression characteristics of these four genes, and salinity stress positively regulates the expression of *SOS* and *NHX1*. In the *VhMYB15*-overexpressing *Arabidopsis*, the expression levels of *AtSOS1/2/3* and *AtNHX1* were significantly higher than those of the control-group *Arabidopsis*. This indicates that *VhMYB15* can maintain the balance of intracellular Na^+^ concentration by positively regulating *AtSOS1/2/3* and *AtNHX1*, thereby enhancing salinity tolerance (Figure 6). This was consistent with the measurement results of electrolyte leakage.

P5CS is a rate-limiting enzyme in the proline biosynthesis pathway, and its mRNA expression level is positively correlated with proline content in plants [48]. There is a high positive correlation between plant stress tolerance and proline accumulation. For example, heterologous expression of the *LcP5CS* gene in *Arabidopsis* enhances plant tolerance to drought stress [49]. The main function of CAT is to remove excess hydrogen peroxide produced during stress and maintain the oxidative balance of plants under adverse conditions [50]. Research has found that under drought stress, the expression level of the CAT gene in drought-resistant varieties is significantly higher than that in drought-sensitive varieties, indicating that these antioxidant enzymes provide innate protection for drought-resistant varieties against ROS toxicity [51]. SnRK2 plays an important role in plant stress tolerance [52]. *AtSnRK2.6* (open stochastic 1) belongs to the *Arabidopsis* SnRK2 family and can be activated and expressed by ABA signaling. Under stress, the protein phosphatase PP2C in plants releases the inhibition of SnRK2.6 protein kinase. Subsequently, SnRK2.6 protein kinase initiates the regulatory effect on downstream signaling components and causes stomatal movement to enhance plant drought tolerance [53]. NCED is a key rate-limiting enzyme in ABA synthesis, widely involved in plant responses to abiotic stress. Overexpression of *AtNCED3* in *Arabidopsis* enhances plant tolerance to drought stress by increasing endogenous ABA levels [54]. Our research results were consistent with the expression characteristics of these four genes, and drought stress positively regulates the expression of *P5CS1*, *CAT1*, *NCED3*, and *SnRK2.6*. In the *VhMYB15*-overexpressing *Arabidopsis*, the expression levels of *AtP5CS1*, *AtCAT1*, *AtNCED3*, and *AtSnRK2.6* were significantly higher than those in the control-group *Arabidopsis*. This indicates that *VhMYB15* can enhance drought tolerance by positively regulating the expression of these stress-related genes, increasing the accumulation of proline and the clearance of ROS (Figure 9).

**Figure 10:**
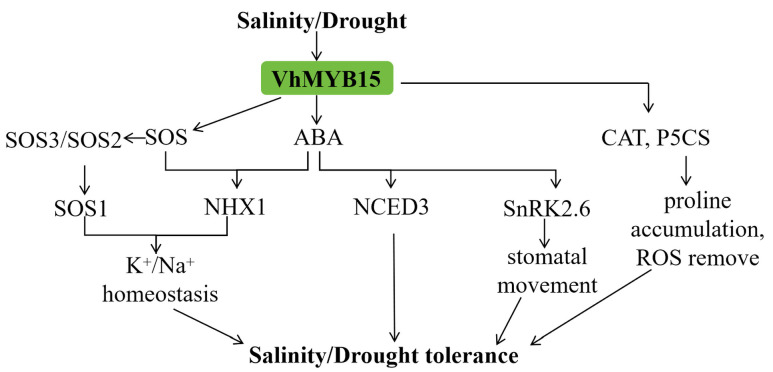
A potential model for salinity or drought adaptation mediated by *VhMYB15*.

In summary, based on the data from this study, we have established a potential stress regulation network model centered on *VhMYB15* (Figure 10). Under salinity stress, the expression of *VhMYB15* activates the SOS pathway, and *AtSOS1* mediates the efflux of Na^+^ in cells under salt stress. Its activity is regulated by the SOS3–SOS2 kinase complex. In addition, the SOS pathway and ABA can regulate the expression of *AtNHX1*, maintain intracellular K^+^/Na^+^ balance and, thus, improve plant salinity tolerance. Under drought stress, *VhMYB15* increases the accumulation of proline and enhances the ability to clear ROS by increasing the expression of *AtP5CS* and *AtCAT1*. In addition, *VhMYB15* responds to drought stress through ABA-dependent pathways. The expression of *VhMYB15* promotes an increase in *AtSnRK2.6* expression, thereby regulating stomatal closure and increasing tolerance to drought stress. *VhMYB15* also increases plant drought tolerance by positively regulating the expression of *AtNCED3*. In summary, overexpression of *VhMYB15* positively regulates plant tolerance to salt and drought stress.

## 4. Materials and Methods 

### 4.1. Plant Materials, Growth Conditions, and Treatments

The ‘Beta’ plants were planted in a light incubator owned by our research group (Laboratory 322, College of Horticulture and Landscape Architecture, Northeast Agricultural University). We selected ‘Beta’ hydroponic seedlings with strong root systems and small growth differences for stress analysis. The specific methods of stresses were as follows: the hydroponic seedlings were placed in Hoagland solution for salinity (Hoagland solution containing 100 mM NaCl, Coolaber, Beijing, China), drought (Hoagland solution containing 20% PEG6000, Coolaber, Beijing, China), cold (Hoagland solution at 4 °C), high temperature (Hoagland solution at 37 °C), and ABA (Hoagland solution containing 100 μM ABA, Coolaber, Beijing, China) treatments [55]. Samples were taken at 0, 1, 2, 4, 8, 12, and 24 h after stresses, and the expression pattern of *VhMYB15* was analyzed using qRT-PCR. *Arabidopsis* was planted in a nutrient bowl (peat soil:vermiculite = 2:1) in the same growth environment as that of ‘Beta’ (humidity: 75%, 25 °C, 16 h of light) [56,57].

### 4.2. Cloning and Bioinformatic Analysis of VhMYB15

Total RNA was extracted from ‘Beta’ leaves and the first cDNA was synthesized. The target gene *VhMYB15* was isolated by a homologous cloning method [55]. Based on the CDS sequence of *VvMYB15* (XM_002285157.4, *Vitis vinifera* L.), Primer 5 was used to design primers for PCR amplification (the primer sequence was as follows: *VhMYB15*-F/R: ATGGTAAGAGCTCCTTGTTG/TCAAAGCTCC TGTAAGCCGC). The primers were from the 5′ to the 3′. The PCR product was connected with the T_5_ cloning vector, and the kanamycin-positive single colony was selected for sequencing, and the CDS sequence of the target gene was obtained [58]. The homology of VhMYB15 was analyzed using NCBI (https://www.ncbi.nlm.nih.gov/, accessed on 21 January 2023) and DNAMAN 5.2, the evolution relationship of VhMYB15 was analyzed using MEGA11, the physicochemical properties of VhMYB15 were analyzed using TBtools v2.042, and the secondary structure and tertiary structure of VhMYB15 were analyzed using SOPMA (http://npsa-pbil.ibcp.fr/cgi-bin/npsa_automat.pl?page=npsa_sopma.html, accessed on 26 March 2023) and SWISS-MODEL (https://swissmodel.expasy.org/, accessed on 26 March 2023) [27,59].

### 4.3. Vector Construction and Subcellular Localization of VhMYB15

The vector pCAMBIA1300 GFP was linearized using restriction endonuclease *BamH*I and *Sal*I [27]. The fusion vector *VhMYB15*–pCAMBIA1300 was obtained by adding 15 bp homologous arms to both ends of the cloned primers (*VhMYB15*-2F/2R: GGTACCCGGGGATCCATGGTAAGAGCTCCTTGTTG/GCTCACCATGTCGACAAG CTCCTGTAAGCCGC; the primers were from the 5′ to the 3′) and connecting the target gene to the linearized vector by a homologous recombination method [6]. The successfully constructed vector was transformed into Agrobacterium GV3101 for subsequent experiments [4].

In order to determine the localization of VhMYB15 in cells, Agrobacterium containing the *VhMYB15*–pCAMBIA1300 vector and pCAMBIA1300 vector were injected into 30-day-old tobacco leaves, and the distribution of GFP fluorescence signals was observed under confocal microscopy. DAPI staining was used as a means of assisting localization [32].

### 4.4. Expression Analysis of VhMYB15

We used the *VvActin-7* gene (*Vitis vinifera,* XM_002282480.5) as an internal reference gene with the following primers: *VvActin-7* F/R: CTTGCATCCCTCAGCACCTT/TCCTGTGGACAATGGA TGGA. The specific primers (*VhMYB15-qF/qR*) were designed to avoid the conservative domain of the CDS region of *VhMYB15*. The specific sequence was as follows: *VhMYB15-qF/qR:* TTGTTGTGATAAGGTGGG/CTTGTTTTGGAAGGGCTC. The primers were from the 5′ to the 3′. The ChamQ Universal SYBR qPCR Master Mix kit (Vazyme, Nanjing, China) was selected for the experiment. We adopted a two-step method for qRT-PCR testing, and the specific method was adapted from Han et al. [60]. It mainly included pre-denaturation (95 °C, 30 s) and a 40-cycle circulation system (95 °C, 10 s; 60 °C, 30 s). The relative expression of *VhMYB15* was calculated by 2^−ΔΔ*C*t^ [60].

### 4.5. Generation of Transgenic Lines 

The Agrobacterium containing the pCAMBIA1300 vector and *VhMYB15*–pCAMBIA1300 vector were activated by a secondary activation method [27]. When the Agrobacterium solution had an OD_600_ = 1, the re-suspension solution was prepared (OD_600_ = 0.5–0.8).

Inflorescences of *Arabidopsis* were soaked in re-suspension solution for 30 s then left to rest for 5 min and cultured in the dark for 24 h. In order to improve the success rate of *Arabidopsis* transformation, each batch of seedlings was infected 2–3 times, with an interval of 1 week between infections. The infected seeds were screened using kanamycin (50 mg L^−1^), and the initially obtained seedlings were T_1_-generation plants, which were further cultured until the seeds were harvested. Subsequently, the expression of *VhMYB15* in T_2_-generation *Arabidopsis* was analyzed by qRT-PCR. In order to obtain homozygous T_3_-generation *Arabidopsis*, the lines with relatively high expression were further cultured. Subsequently, salinity and drought treatments were carried out using T_3_-generation *Arabidopsis* [21]. 

### 4.6. Analysis of Related Physiological Indexes in VhMYB15-Overexpressing Arabidopsis

WT, UL, and transgenic *Arabidopsis* lines (L1, L3, and L7) with similar growth were selected to measure the stress-related physiological indexes. Each *Arabidopsis* line was divided into three equal parts: one part was cultured under normal conditions, and the other two parts were treated with salinity stress or drought stress. The specific method was irrigation with Hoagland solution containing 200 mM NaCl for 8 d under salinity stress and no watering for 10 d under drought stress. The survival rate was calculated after 3 d of stress relief. The phenotypes of the plants before and after stresses were observed, and the related physiological indexes of the plants were measured. The chlorophyll content was measured by the anhydrous ethanol extraction method, the proline was extracted by sulfonic salicylic acid, and the color reaction was carried out with ninhydrin [50]. Malondialdehyde was extracted by thiobarbituric acid [3]. The activities of peroxidase (POD), superoxide dismutase (SOD), and catalase (CAT) were detected with the Suzhou Grace Biotechnology test kit (item No. G0107F, G0101F, G0105F, Comin, Suzhou, China).

### 4.7. Expression Analysis of Stress-Related Genes in VhMYB15-Overexpressing Arabidopsis

The RNA of *Arabidopsis* lines (WT, UL, L1, L3, L7) was extracted under salinity stress (200 mM NaCl for 8 d) and drought stress (no watering for 10 d), and cDNA was obtained by reverse transcription. The expression of *AtNCED3*, *AtSnRK2.6*, *AtP5SC*, *AtCAT1*, *AtSOS2*, *AtSOS3*, *AtSOS1*, and *AtNHX1* downstream genes of *VhMYB15* was detected by qRT-PCR. *AtActin2* was selected as the internal reference gene. The specific primers were as follows: *AtP5CS1*-F/R: AGGGAAAGTTCCA GAAAG/CATAACTAAGCGAGCCAC; *AtCAT1*-F/R: GTCCTGGGATTCAGACAGGC/GGCC TCACGTTAAGACGAGT; *AtNCED3*-F/R: TTGATGCTCCAGATTGCTTC/GGACCCTATCACG ACGACTT; *AtSnRK2.6*-F/R: AGATCCCGAGGAACCAAAGA/CTCTTTGCAGGGTCAGCA AC; *AtSOS2*-F/R: GCAAGGGAAGAAGAAGAAGT/TCTCCGCTACATAACTGCC; *AtSOS3*-F/R: GAATCCATCGCTCATCAA/CCATTTCTTCCTCTTCACA; *AtSOS1*-F/R: TTCATCATCCTC ACAATGGCTCTAA/CCCTCATCAAGCATCTCCCAGTA; *AtNHX1*-F/R: AGCCTTCAGGGAA CCACAAT/CTCCAAAGACGGGTCGCATG; *AtActin2*-F/R: TTACCCGATGGGCAAGTCA/AAACGAGGGCTGGAACAAGA [61]. The primers were from the 5′ to the 3′. The PCR program and expression calculation method were the same as those in Section 4.4.

### 4.8. Statistical Analysis

Using GraphPad principle (v8.0.2.263) software, physiological index data of various *Arabidopsis* lines (WT, UL, L1, 3, 7) before and after stresses were analyzed, and standard error (±SE) and differences (* *p* ≤ 0.05, ** *p* ≤ 0.01) were marked. Both biological and technical replicates were performed three times.

## 5. Conclusions

*VhMYB15*, an R2R3-type MYB TF isolated from ‘Beta’, can respond to various stresses, with higher expression levels in its roots and mature leaves under salinity and drought stresses. By constructing *VhMYB15*-overexpressing *Arabidopsis* and analyzing its phenotype and stress-related physiological indicators, it was found that heterologous expression of *VhMYB15* improved the survival ability of *Arabidopsis* under salinity and drought. In addition, the expression level analysis of MYB downstream stress-related genes also confirmed this result. The analysis of *VhMYB15* function can provide a possibility for the molecular breeding of grapes.

## Data Availability

The original data for this present study are available from the corresponding authors.

## Supplementary Materials

The following supporting information can be downloaded at: https://www.mdpi.com/article/10.3390/ijms25031534/s1.
