# Supplementary material for: Overexpression of a ‘Beta’ MYB Factor Gene, VhMYB15, Increases Salinity and Drought Tolerance in Arabidopsis thaliana
Source: Int J Mol Sci. 2024 Jan 26;25(3):1534. doi: 10.3390/ijms25031534 (PMC10855843; doi:10.3390/ijms25031534)
Supplement: Supplementary file 1 [file ijms-25-01534-s001.zip › ijms-2808677-supplementary.pdf]

```
1   ATGGTAAGAGCTCCTTGTTGTGATAAGGTGGGATTGAAGAAGGGGCCATGGACGACTCAGGAAGATCAAATTTTGACGGCCTACGTTCTACAACAT
1    M  V  R  A  P  C  C  D  K  V  G  L  K  K  G  P  W  T  T  Q  E  D  Q  I  L  T  A  Y  V  L  Q  H

97  GGCCATGGAAACTGGCGAGCCCTTCCAAAACAAGCCGGATTACTAAGATGTGGGAAGAGTTGCAGACTCCGATGGATAAACTACTTACGACCTGAT
33   G  H  G  N  W  R  A  L  P  K  Q  A  G  L  L  R  C  G  K  S  C  R  L  R  W  I  N  Y  L  R  P  D

193 ATCAAGAGAGGAAATTTCAGCAGAGAAGAAGAGGATACCATCATTGAGTTACATGAAATGCTTGGCAATAAATGGTCGGCCATAGCAGCCAGCCTC
65   I  K  R  G  N  F  S  R  E  E  E  D  T  I  I  E  L  H  E  M  L  G  N  K  W  S  A  I  A  A  S  L

289 CCGGGTCGGACCGACAACGAGATAAAAAATGTCTGGCACACACACCTGAAGAAGAGGCTCAAAAAAAAATTGGCCACACCGGATTCCAAAGGCCAC
97   P  G  R  T  D  N  E  I  K  N  V  W  H  T  H  L  K  K  R  L  K  K  K  L  A  T  P  D  S  K  G  H

385 TTTACTGCAGCAGCTTCCACATGTGACTCGGATTCTTTTAATTCCCCCGGAAAAATGTCCCCTCAACCGTCCTCCAGTGAATTCTCTTCATTTACA
129  F  T  A  A  A  S  T  C  D  S  D  S  F  N  S  P  G  K  M  S  P  Q  P  S  S  S  E  F  S  S  F  T

481 GACTCCTCAACAAGGACCATGGAAACACACAGCACTGGCGTCAAGAATGAACAGATGGAAGAGTACTCAACGGAATCATTTCCTGAAATCGATGAA
161  D  S  S  T  R  T  M  E  T  H  S  T  G  V  K  N  E  Q  M  E  E  Y  S  T  E  S  F  P  E  I  D  E

577 AGTTTTGGTCAGACGCGCTGTCCTCTGATAATTCAAGCACGGCGTCAGATTTCCCAGCAGTCGCAGACGAATTACAGCTTCAGACACCGGAGCTC
193  S  F  W  S  D  A  L  S  S  D  N  S  S  T  A  S  D  F  P  A  V  A  D  E  L  Q  L  Q  T  P  E  L

673 GCCTATGGACAAATTTCAAACATGATGGATGATGGCATGGAGTTTTGGTATGACGTTTTCATCAGAGCTGGCGGCTTACAGGAGCTTTGA
225  A  Y  G  Q  I  S  N  M  M  D  D  G  M  E  F  W  Y  D  V  F  I  R  A  G  G  L  Q  E  L  *
```

Figure S1. Nucleotide and amino acid sequence of *VhMYB15*. The red underlined part was the R-repeated conserved domain.

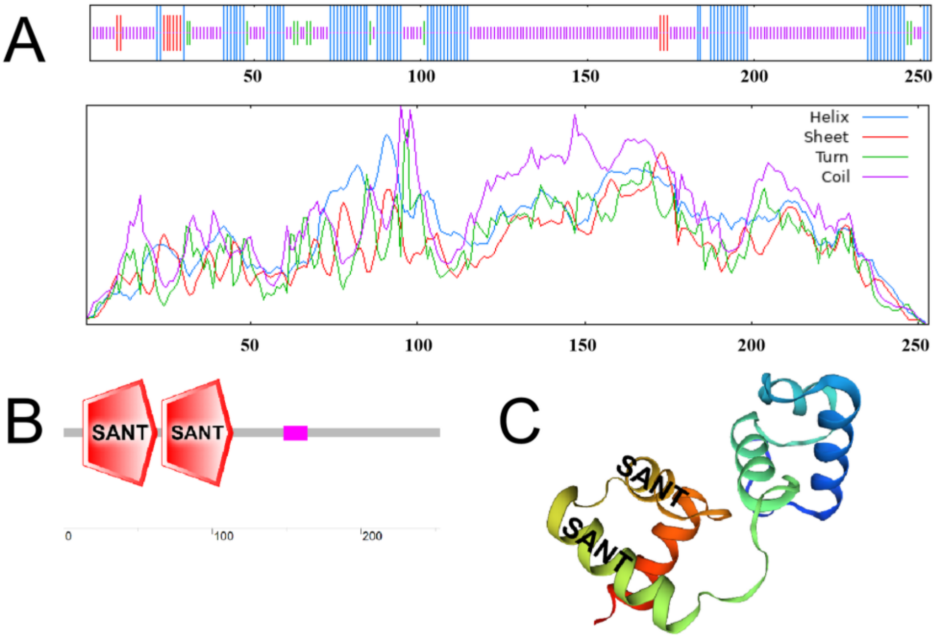

Figure S2. Structure analysis of VhMYB15 protein. (**A**) The secondary structure; (**B**) functional domain; (**C**) tertiary structure of VhMYB15.
